# Effects of Passive Hydrotherapy WATSU (WaterShiatsu) in the Third Trimester of Pregnancy: Results of a Controlled Pilot Study

**DOI:** 10.1155/2015/437650

**Published:** 2015-03-01

**Authors:** Agnes M. Schitter, Marko Nedeljkovic, Heiner Baur, Johannes Fleckenstein, Luigi Raio

**Affiliations:** ^1^Department of TCM/Acupuncture, Institute of Complementary Medicine IKOM, University of Bern, Imhoof-Pavillon, Inselspital, 3010 Bern, Switzerland; ^2^Department of Health, Bern University of Applied Sciences, Murtenstrasse 10, 3008 Bern, Switzerland; ^3^Department of Obstetrics and Gynecology Inselspital, University Hospital of Bern, Effingerstrasse 102, 3010 Bern, Switzerland

## Abstract

*Background*. WATSU (WaterShiatsu) is a complementary therapeutic treatment method comprising passive stretches and massage techniques administered in 35°C warm water. Pregnant women claim safe methods to reduce pain, stress, and fatigue. Therefore, we conducted a pilot study evaluating the effects of WATSU on pregnancy-related complaints in third trimester pregnant women. *Methods*. Nine healthy pregnant women at gestational week ≥34 were included in an intervention group (receiving WATSU) and compared to eight women in a passive control group (receiving no treatment). WATSU was performed on days 1 and 4 of the study, accompanied by ultrasound examinations. Outcomes include physiological and psychometric as well as qualitative data. Participants in the control group completed questionnaires only. *Results*. WATSU was found to significantly lower participants' levels of stress and pain and to improve their mental health-related quality of life and mood. In comparison to the passive control group, participants in the intervention group reported reduction in perceived stress from day 1 to day 8 (*P* = 0.036, Cohen's *f* = 0.57). Qualitative data indicate that WATSU was appreciated as enjoyable and deeply relaxing. No negative side effects were reported. *Conclusion*. Our findings support the notion that WATSU yields therapeutic benefits for pregnant women and warrant further research. This study has been registered at ClinicalTrials.gov: NCT01708018.

## 1. Introduction

Several researchers propose that, during pregnancy, maternal mental and physical wellbeing are transferred to the fetus resulting in epigenetic changes implicating consequences for a lifetime [1–4]. Hence, with respect to the child's future development, severe maternal stress and distress cannot be considered to be of transient nature [5]. Moreover, maternal effect and attitude during pregnancy are also predictive factors for postnatal depressive symptoms [6], which in turn are likely to affect the child's wellbeing.

WATSU (an acronym based on WAter and shiaTSU) is a body-based method [7] comprising buoyancy, passive stretches, and massage techniques, including massage and palpation of acupuncture points that is administered in warm water. WATSU has been described as applicable during pregnancy [8], where it is claimed to reduce pregnancy-related low back pain, to relax hypertonic muscles including those of the uterus, to improve the overall sense of wellbeing, and to deepen the relationship of the mother with her unborn child.

In the 1980s, WATSU was created by massage-therapist Harold Dull, whose personal affinity to water led him to practice Masunaga-Shiatsu (also known as Zen-Shiatsu) in water heated to roughly skin temperature (308.15 Kelvin; 35°C, 95°F). He used Shiatsu massage of acupuncture points, joint mobilization, and tissue stretches and added gentle massage techniques to harmonize the energy flow (qi, 氣) according to the principles of Traditional Chinese Medicine (TCM), from which Shiatsu originates [9]. The increased clinical implementation of WATSU in interdisciplinary treatment settings such as rehabilitation facilities indicates a growing acceptance of this body-based complementary therapeutic intervention; it is used as a component in multimodal treatment settings focusing on posttraumatic stress disorder, and anxiety [10, 11], chronic pain and fibromyalgia [11, 12], stress-related illnesses [13], depression [11, 14], and sexual dysfunction [15]. WATSU has also been recommended as a treatment for patients with hemiparesis, multiple sclerosis, cerebral palsy, and spinal cord injury [16, 17]. Considering physiological effects on the cardiopulmonary system due to physical exposure to hydrostatic pressure, certain cardiac conditions, for example, chronic heart failure [18] and respiratory impairments such as cystic fibrosis [19] can be regarded as potential indications for this treatment.

However, clinical trials investigating therapeutic effects of WATSU are still scarce. To our knowledge, to date, only three small scale trials on WATSU have been published [12, 16, 20].

Regarding pregnancy, gymnastics in water were observed to have pain-relieving effects [21, 22], as were massage [23] and birth in water [24]. Active hydrotherapy during pregnancy is considered to be beneficial and safe [25], while the effects of passive hydrotherapy during pregnancy so far have not been the subject of scientific investigation. Therefore, we designed a pilot study to examine safety of WATSU as well as a broad range of issues to identify those worthwhile pursuing in future research, that is, potential therapeutic effects of WATSU on self-reported stress, pregnancy-related pain, mood and quality of life, amount of amniotic fluid, blood flow characteristics, spontaneous course of breech presentations, and prospects of external cephalic versions in third trimester pregnant women. Additionally, qualitative data reflecting participants' perception of the intervention were assessed and analyzed.

## 2. Methods

### 2.1. Study Design

We conducted a controlled clinical pilot study at the Department of Obstetrics and Gynecology at Bern University Hospital investigating the effects of WATSU on pregnant women with pregnancy-related complaints at week ≥34 of gestation. The research protocol was formally approved by the Ethics Committee of the Canton of Bern, Switzerland, and carried out according to the Helsinki Declaration. Recruitment was carried out from May 2012 to May 2014 by medical staff at Bern University Hospital, as well as through advertisements via the midwifery association of the Canton of Bern. Potential participants were provided with complete written and verbal information about the study and written consent was obtained prior to participation. Interested healthy women with a singleton pregnancy in the 34th or greater week of gestation underwent a telephone-screening. Exclusion criteria were any pathological findings during pregnancy, neurological deficits resulting from low back pain, WATSU-treatment within the past four weeks, and poor language skills. Women reporting a breech presentation were included in the study if they did not plan external cervical version. Participants in the intervention group received standardized WATSU treatments at days 1 and 4, accompanied by ultrasound examinations before and after WATSU treatment and on day 8. Participants were allocated to the passive control group if they refused to undergo intervention or ultrasound examinations, or if they lived too far from the site of the intervention. If allocated to the passive control group, participants did receive neither WATSU nor any alternative treatment on the part of the study. All participants were free to maintain additional medical and/or therapeutic treatments during the study. Assessments took place on days 1 (baseline), 4, and 8 (follow-up). Participants were not financially compensated; participants in the control group were offered a free WATSU-treatment after study completion.

The study has been registered at ClinicalTrials.gov: NCT01708018.

### 2.2. Intervention

WATSU treatments were performed by four specialized therapists with ten to sixteen years of professional experience and certification by the Swiss Aquatic Bodywork Association (Netzwerk für Aquatische Körperarbeit (NAKA)). Each participant in the intervention group received a standardized WATSU-treatment on the first and fourth day of study participation, administered by the same therapist. Participants were free to choose a female or a male therapist. Sessions lasted 60 minutes and took place in the morning from 9:00 a.m. to 10:00 a.m. in a therapy pool located at the University Hospital of Bern filled with 35°C fresh water. The administered motion sequence “WATSU-Transition-Flow” [9] was adapted for women in their third trimester of pregnancy and was followed closely. During the WATSU-treatment, participants' abdomens were not touched. Deviations from the standardized treatment protocol were documented for each treatment session. Each session started with a brief verbal description of the procedure and ended with the opportunity for the client to give verbal feedback. During WATSU treatment, participants rested in a supine position, predominantly being supported at the back of their head and at their pelvis or knees by their therapist's forearms. To unburden participants' lower backs, floating devices were attached to their thighs. In the course of treatment, therapist and participant were in continuous physical contact with distances varying from a full arm's length to cradled positions. The participant was slowly floated back and forth through the water in large circular patterns, generated by the therapist's rotation around her/his own body axis. Following the session, participants were asked to drink 500 mL of water to compensate for body fluid loss due to increased diuresis [26, 27]. If indicated and with participants' consent, external cephalic version (ECV) was performed on day 9 of the study. Participants in the passive control group received neither WATSU nor an alternative treatment in the context of this trial.

### 2.3. Outcome Measures

Sociodemographic data (age, height, weight, prior deliveries, week of gestation, and fetal position) and baseline data (perceived stress, pain, and quality of life) were assessed on day 1.

#### 2.3.1. Baseline Assessment on Day 1 and Follow-Up Assessment on Day 8

Included the Perceived Stress Scale (PSS) and theMedical Outcomes Study 36-Item Short Form Health Survey (SF-36). Both questionnaires referred to participants' self-reported condition in the past week.

*Perceived Stress Scale (PSS)*. This 10-item questionnaire assesses participants' cognitive evaluation of stress perception [28]. Participants estimate how unpredictable, uncontrollable, and overloaded they perceive their lives to be on a 5-point scale ranging from “never” to “very often.” Good internal consistency is reported (Cronbach's *α* = 0.87) [29]. 

*Medical Outcomes Study 36-Item Short Form Health Survey (SF-36)*. Health-related quality of life was assessed using a questionnaire comprising eight subscales: “physical functioning,” “role physical,” “bodily pain,” “role emotional,” “vitality,” “mental health,” “general health perception,” and “social functioning.” The SF-36 shows good internal consistencies with Cronbach's *α* > 0.70 in all subscales except “general health perception” (0.57) and “social functioning” (0.69) [30].

#### 2.3.2. Psychometric Assessment on Days 1 and 4 Immediately before and after WATSU Treatments

Included Visual Analogue Scales (VAS) assessing pain and stress and the Multidimensional Mood Questionnaire (MDMQ). These instruments referred to participants' self-reported actual condition. 

*Stress and Pain Related Visual Analogue Scales (VAS)*. Visual Analogue Scales have a length of 100 mm, with 0 mm indicating no stress, respectively, no pain at all, and 100 mm representing maximal perception of stress, respectively, high pain. VAS scales proved to be valid and reliable [31, 32]. 

*Multidimensional Mood Questionnaire (MDMQ)*. The validated mood questionnaire with good internal consistencies (Cronbach's *α* between 0.73 and 0.89) assesses treatment-related changes in self-reported mood. It consists of a list of 12 adjectives that address current mood, calmness, and alertness (e.g., “happy,” “nervous,” and “awake”) ranked on a 5-point scale ranging from “not at all” to “very much.” The sum score ranges from 12 to 60 with higher scores indicating better mood [33].

#### 2.3.3. Ultrasound Examination

All ultrasound examinations were carried out twenty minutes before and after WATSU treatment, as well as in the morning of day 8 by gynecologists using Voluson E8 Expert, GE Medical Systems (Zipf, Austria), with curved array transducer of 5–8 MHz. Ultrasound measurements included examination of fetal position and assessment of amniotic fluid volume, measuring the single deepest amniotic fluid pocket (SDP) [34, 35]. Doppler examination of the umbilical artery and both uterine arteries was performed. Blood flow characteristics were assessed measuring the pulsatility index (PI). Along with this assessment, the tonus of the uterus was palpated by the examining gynecologist.

#### 2.3.4. Qualitative Outcome Measures

Participants in the intervention group were asked to fill out a qualitative questionnaire answering the following questions right after their second WATSU-treatment: “How was your experience being treated with WATSU?” “Which changes did you notice in response to your WATSU treatment?” “Which aspects of your WATSU treatment were less pleasant for you?” and “Do you have any suggestions for improvement?”

### 2.4. Data Analysis

Quantitative data were analyzed based on intention to treat. For missing data, the last value was carried forward. Quantitative data analyses were conducted using SPSS (version 19) statistical software package for Windows (IBM SPSS Statistics, Somers, NY, USA). Prior to statistical analyses, all data were tested for homogeneity of variance and normal distribution employing Levene and Kolmogorov-Smirnov tests. Between-group differences in sociodemographic characteristics, baseline values, and mean change values of outcome measures from baseline to follow-up assessment were analyzed using Mann-Whitney *U* test for continuous data, while the evaluation of categorical data was based on visual inspection. Analyses of outcome measures within the intervention group were performed using Wilcoxon test. All analyses were two tailed, with the level of significance set at *P* < 0.05 with 95% confidence interval. All continuous data are presented as mean value ± standard deviation (SD). For the purpose of international comparability, outcome values of the SF-36 main scales were standardized by employing the weighting coefficient for US population and transformed into percentages [30]. Effect size parameters (*f*) were derived from partial *η* ^2^ values and were reported based on the following effect size conventions: *f*: 0.10 = small, 0.25 = medium, and 0.40 = large [36].

Narrative questionnaire data reflecting participants' experiences with WATSU were systematically organized into analytical units, which were inductively classified into thematic subcategories and main categories according to the Mayring-triangulation-model [37]. All generated categories were quantitatively described by indicating frequency of mentions in absolute and percentage values. Qualitative data reflecting context-related information were narratively summarized.

## 3. Results

**Figure 1:**
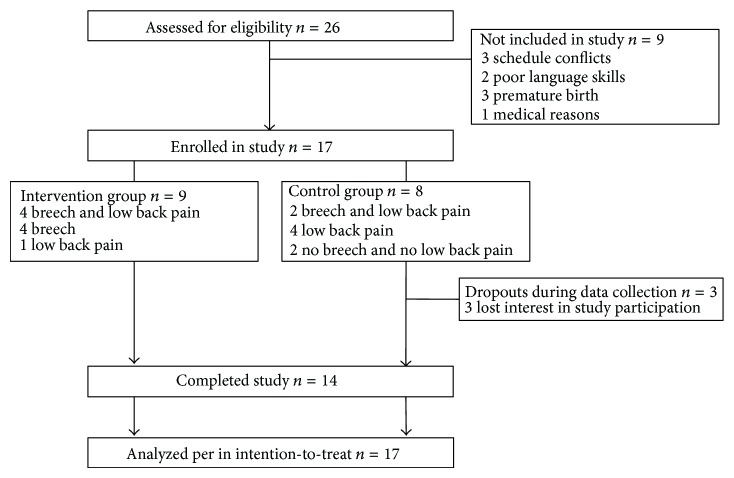
Consort flow diagram.

Seventeen of the 26 recruited women were included with nine participants being allocated to the intervention group and eight to the control group. Three participants in the control group were lost to follow-up (for details see Figure 1). Finally, data from all seventeen study participants were analyzed according to the intention-to-treat principle. No adverse events were reported, and all of the WATSU-treatments were carried out as scheduled.

Group and baseline characteristics did not differ significantly between the two study groups (see Table 1). Study participants were between 27 and 40 years of age (32.0 ± 3.2) and 69% of them were primiparous.

### 3.1. Quantitative Results

As presented in Table 2, analyses within the intervention group revealed a significant improvement in mental health-related quality of life (SF-36: *P* = 0.018) and a significant reduction in perceived stress (PSS: *P* = 0.027) from baseline to follow-up assessment.

**Figure 2:**
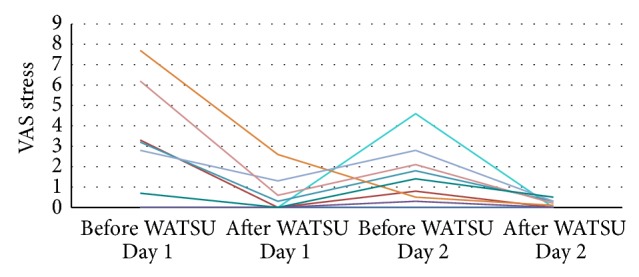
Changes in perceived stress measured by VAS (Visual Analog Scale; higher scores indicate increased stress, maximum score: 10).

Analyses examining immediate effects of the two WATSU-treatments on participants' level of stress, pain, and mood consistently showed significant improvements in all outcome measures (see Table 3; for individualized stress curves see Figure 2).

In contrast to the control group, participants in the intervention group reported significant decreases in perceived stress from day 1 to day 8 whereas no significant group differences were found for mean change values of SF-36 main scales (see Table 4). Short term effects of WATSU on stress, pain, and mood that were observed within the intervention group were also present in comparison to the control group (see Table 5).

**Figure 3:**
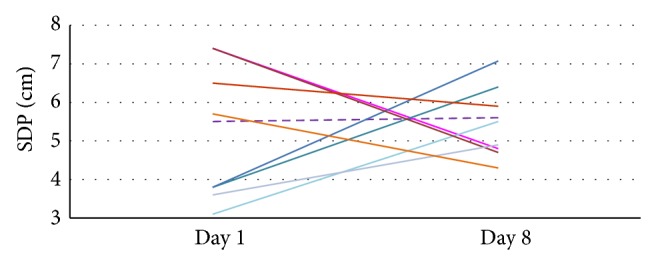
Changes in the amount of amniotic fluid in centimeter in the intervention group measured at the SDP (single deepest amniotic fluid pocket).

Data from ultrasound examinations in the intervention group revealed normal amounts of amniotic fluid with respect to the gestational age, that is, >2 cm and <8 cm at the single deepest amniotic fluid pocket (SDP) [38, 39] at baseline, before and after each WATSU treatment, and at follow-up (day 8) for all participants of the intervention group. Four participants with low baseline values (≤3.8 cm, mean 3.6 cm ± 0.1 cm) showed an average increase in amniotic fluid to 5.9 cm ± 1.5 cm on day 8. For one participant, the values remained practically unchanged (from 5.5 cm to 5.6 cm), and in four cases that presented values ≥5.6 cm (6.8 cm ± 0.8 cm) at baseline, the amniotic fluid value decreased to a mean value of 5.02 cm ± 0.7 cm (see Figure 3).

Similarly, the pulsatility indexes (PI) obtained from the umbilical artery and from both maternal vessels were within normal ranges for the respective gestational age and no significant changes were observed comparing pre- and posttreatment values (*P* values ≥ 0.23). No signs of adverse reactions were detectable in ultrasound measures at any point in the study.

Due to optimal tonus of the uterus, four of the participants in the intervention group underwent cephalic version on day 9 of the study, with two attempts being successful. Of the eight fetuses in breech position in the intervention group, one spontaneously presented in cephalic position on day 4. The two fetuses in breech in the control group remained in this position up to day 8 and one attempt for cephalic version failed.

### 3.2. Qualitative Results

A total of 57 comments reflecting subjective perceptions of the intervention were obtained from nine participants and were classified into main and subcategories as presented in Table 6.

Relaxation was most frequently reported as a major overall impression of the WATSU-treatments but was also distinctly mentioned by participants as an effect of treatment on their physical and mental states. Participants shared observations regarding body-related changes, for example, regained mobility and flexibility and decreased pain. They empathically reflected on how this experience might be perceived by their unborn children and on the nature of jointly experiencing an aquatic environment. Some of the women expressed gratitude for having been able to participate in the study and emphasized their impressions of having learned something about relaxation and surrendering that they perceived as a very helpful preparation for the upcoming birth. The intervention was referred to using positive descriptions such as “*like a dream*.” Mind-related aspects represented the majority (26.3%) of comments. All participants described WATSU as an experience they had fully enjoyed, and the opportunity to suggest improvements was instead used to emphasize satisfaction by six participants. The four stated suggestions concerning technical and process-related aspects of the study involved timing issues such as delays in ultrasound examination as well as a lack of clarity about where to hand in feedback-forms. One person mentioned a WATSU treatment-specific observation (having felt uncomfortable with hip flexion), and one woman suggested starting WATSU treatment earlier in pregnancy.

## 4. Discussion

Active aquatic therapies have been studied extensively and proven to be beneficial for pregnant women [25]. Since passive hydrotherapy was not yet scientifically investigated with respect to its effects on pregnant women, we conducted a controlled pilot study to investigate the effects of WATSU on women in their ≥34th week of gestation, as well as to evaluate which of the investigated parameters would be suitable outcome measurements for further large-scale clinical trials.

As the impacts of maternal stress on unborn children can be severe [1–5], interventions that effectively reduce stress during pregnancy are desirable. Significant short-term improvements measured with VAS and MDMQ and significant medium-term decreases in perceived stress (PSS), with respective increases in the mental component of SF-36 main scale, indicate an optimized situation concerning the emotional wellbeing of the participants in the intervention group. Components of WATSU including gentle touch are believed to act in a stress reducing manner [40, 41]. In addition, the therapist's thoroughly compassionate attitude allows her/him to enter a parasympathetic state that the patient is nonverbally encouraged to join in [42]. During immersion, patients experience decreased heart rates [18] that are organically anticipating and promoting a parasympathetic state of relaxation. Reduced hypothalamic-pituitary-adrenal axis activity, that is, lower plasma cortisol levels along with increased mental and physical relaxation in context with immersion, has previously been reported [43, 44]. A potential mode of action promoting emotional wellbeing might be the activation of afferent C-tactile fibers during immersion. It has been proposed that these fibers transmit slow gentle touch—analogous to bypassing water—that has been observed to activate emotional brain areas [45, 46].

Short-term effects on pain were significant in our study (see Tables 3 and 5). This is of clinical relevance as epidemiological data indicate that two out of three pregnant women suffer pregnancy-related low back pain [22]. During pregnancy, it is recommended to refrain from analgesics [47], and thus nonpharmacologic alternatives are desirable. Prior research found that merely being immersed in warm water decreases pain [44, 48]. Passive hydrotherapy offers in addition unique possibilities of weightless mobilization in a quasi gravity-free environment with reduced joint compression forces. This in turn creates turbulence and currents causing a sensory overflow, which is regarded as one mechanism in pain reduction following immersion [17]. According to expert opinion, WATSU might be alleviating excessive muscle tone and pain due to rotational movements of the trunk and gentle rocking of the whole body, leading to dampened muscle tone as a side effect of vestibular system activation [49].

Neither the amniotic fluid volume nor umbilical blood flow characteristics were significantly altered after WATSU treatment. Although the sample size is small and therefore generalizability of the results has to be taken carefully, no adverse maternal and fetal reactions have been observed. The examining gynecologists perceived the tone of the uterus as being lower after WATSU treatment, encouraging them to emphatically suggest external cephalic version in four cases. Their observations might be evaluated objectively by electromyogram (EMG) in future studies. Breech presentation is a complication that occurs in only 3-4% of pregnancies but is a very real threat to the mother's as well as the child's life [50]. External cephalic version to correct the child's position is being suggested although associated with risks for mother and child [51, 52]. The success rate of external cephalic version attempts is strongly dependent on uterine tension (OR 18; 95% CI, 12–29) [53] and to a lesser extent also on the amount of amniotic fluid [54].

Only 10% of the amniotic fluid is regulated by transmembranous processes while intra-amniotic interactions—mainly fetal urine production, swallowing, and fluid secretions from the respiratory tract—regulate 90% [55, 56]. However, four participants showed slight increases in SDP measurements from day 1 to day 8. We also measured decreases in amniotic fluid over the period of seven days, which stands in contrast to the findings described in the literature [55, 57]. Since no formalized guidelines for the management of polyhydramnion exist to date [38], the decrease of amniotic fluid due to immersion might be a finding warranting further exploration. While short-term changes in the amount of amniotic fluid due to immersion were observed to be equalized within 30 minutes after immersion [58], the changes observed in our study took place over a longer period of time and would therefore suggest the involvement of respective regulatory processes.

Perceived relaxation and pleasantness seem to be among the most obvious features of passive hydrotherapy and were mentioned by participants in this study with great consistency in qualitative feedback. Interestingly, WATSU, despite its massage character, was attributed to mental effects most frequently. It appears to have been perceived by our participants rather as a mind-body-intervention, particularly suitable to inviting serenity as well as broadened awareness and mindfulness. Comments suggest usefulness of WATSU not only at the third trimester of pregnancy, but also in earlier stages, for example, potentially supporting women in overcoming ambivalence with respect to their motherhood.

## 5. Limitations

The following limitations of this study need to be addressed. The original study design implied ultrasound examinations on the control group as well to assess the natural course of changes in the amount of amniotic fluid [59]. In fact, only one woman in the control group agreed to follow this procedure. Also blinding of any party but the statistician is a challenge.

Most of the mothers-to-be in our intervention group learned about the diagnosis of breech presentation a short time before their enrollment and were thus suddenly being confronted with decisions about whether or not to undergo external cephalic version or cesarean section. In this respect, they differed considerably from participants in the control group, who represented a more general population. It is therefore unclear, to what extent specifically the medium-term effects of emotional recovery can be attributed to the treatment.

A small sample size may lead to the identification of large, clinically relevant effects; however, selection bias and overly weighted outliers cannot be ruled out. Hence, caution is appropriate when interpreting the present results. Due to the small sample size, we were not able to definitively interpret all of our findings scientifically, particularly the impact of WATSU treatment on spontaneous versions of children in breech position.

In qualitative data, saturation might have been achieved in terms of categories; however, since several aspects were mentioned only once, there might have been additional new statements concerning the experience, had more individuals participated in the survey.

## 6. Conclusions

To our knowledge, this is the first clinical study investigating the effects of passive hydrotherapy during pregnancy. It demonstrates significant benefits of WATSU with respect to stress, pain, mood, and mental health-related quality of life. Apparently, lowered tone of the uterus encouraged attempts of cephalic versions when children were in breech position. The treatment was described as very agreeable by the participants and appears to be a safe intervention.

These findings support the notion that WATSU yields therapeutic benefits for pregnant women; therefore, its integration into interdisciplinary treatment approaches should be considered. On the basis of this pilot study, larger trials should be established to further investigate and confirm the impact of the observed effects.

## Figures and Tables

**Table 1:** Group and baseline characteristics.

Characteristics^a^	Intervention group (*n* = 9)	Control group (*n* = 8)	Complete sample (*n* = 17)	*P* ^b^
Age (years)	31.3 (±2.7)	32.8 (±3.7)	32.0 (±3.2)	0.56
BMI (kg/m^2^)	27.5 (±3.4)	25.5 (±2.3)	26.6 (±3.1)	0.31
Primiparous (yes/no)	6/3	7/1	13/4	n/a
Week of gestation	36.3 (±0.7)	35.8 (±1.2)	36.1 (±1.0)	0.43
Breech presentation (yes/no)	8/1	2/6	9/8	n/a
PSS score	14.8 (±5.0)	14.8 (±11.7)	14.8 (±8.5)	0.81
SF-36 main scales				
(i) physical component	47.0 (±6.8)	40.4 (±10.7)	43.9 (±9.2)	0.18
(ii) mental component	46.4 (±11.6)	52.8 (±6.9)	49.4 (±9.9)	0.12

^a^All continuous data are presented as mean (±SD).

^b^ *P* values refer to Mann-Whitney *U* test.

n/a, not applicable; BMI, body mass index; PSS, Perceived Stress Scale; SF-36, Medical Outcomes Study 36-Item Short-Form Health Survey.

**Table 2:** Analyses of medium-term changes in psychometric outcome measures within the intervention group.

Variables^a^	At baseline (day 1)	At follow-up (day 8)	Δ value	*P* ^b^
PSS score^c^	14.8 (±5.0)	12.7 (±5.3)	−2.1 (±2.2)	0.027^*^
SF-36 main scales^d^				
(i) Physical component	47.0 (±6.8)	45.3 (±11.0)	−1.7 (±6.7)	0.735
(ii) Mental component	46.4 (±11.6)	50.0 (±12.3)	+3.3 (±4.0)	0.018^*^
SF-36 sub scales^d^				
(i) Physical function	58.9 (±11.4)	61.1 (±18.7)	+2.2 (±10.9)	0.527
(ii) Role physical	72.2 (±49.1)	66.7 (±43.3)	−5.6 (±48.1)	1.00
(iii) Bodily pain	72.7 (±49.2)	70.4 (±26.3)	−2.2 (±4.1)	0.157
(iv) General health	81.3 (±10.8)	79.7 (±17.4)	−2.4 (±13.8)	0.786
(v) Vitality	50.6 (±18.3)	55.6 (±18.3)	+5.0 (±9.0)	0.114
(vi) Social function	76.4 (±22.1)	79.2 (±21.7)	+2.8 (±8.3)	0.317
(vii) Role emotional	63.0 (±38.9)	70.4 (±38.9)	+7.4 (±22.2)	0.317
(viii) Mental health	67.1 (±16.9)	72.0 (±18.6)	+4.9 (±8.4)	0.125

^a^All continuous data are presented as mean (±SD).

^b^ *P* values refer to Wilcoxon test. ^*^ *P* > 0.05; CI 95%.

^c^PSS, Perceived Stress Scale; positive mean change values represent an increase in perceived stress.

^d^SF-36, Medical Outcomes Study 36-Item Short-Form Health Survey; positive mean change values represent an increase in health related quality of life.

**Table 3:** Analyses of short-term changes in psychometric outcome measures within the intervention group.

Variables^a^	Before WATSU	After WATSU	Δ value	*P* ^b^
VAS stressfulness (mm)^c^				
(i) At day 1	27 (±28)	5 (±9)	−21 (±22)	0.028^*^
(ii) At day 4	16 (±15)	2 (±2)	−14 (±14)	0.012^*^
VAS pain (mm)^c^				
(i) At day 1	9 (±12)	2 (±3)	−8 (±9)	0.028^*^
(ii) At day 4	12 (±10)	1 (±1)	−11 (±11)	0.012^*^
MDMQ mood scale score^d^				
(i) At day 1	16.9 (±2.8)	18.7 (±2.6)	+1.8 (±1.9)	0.042^*^
(ii) At day 4	14.7 (±6.8)	19.3 (±1.0)	+4.7 (±6.2)	0.027^*^
MDMQ alertness scale score^d^				
(i) At day 1	13.9 (±3.2)	16.0 (±2.4)	+2.1 (±3.6)	0.122
(ii) At day 4	10.7 (±5.0)	16.3 (±2.4)	+5.7 (±4.7)	0.007^*^
MDMQ calmness scale score^d^				
(i) At day 1	13.7 (±3.0)	18.0 (±2.9)	+4.3 (±3.0)	0.012^*^
(ii) At day 4	12.8 (±5.6)	18.3 (±1.7)	+5.6 (±5.8)	0.015^*^

^a^All continuous data are presented as mean (±SD).

^b^ *P* values refer to Wilcoxon test. ^*^ *P* > 0.05; CI 95%.

^c^VAS, Visual Analog Scale; positive mean change values represent an increase in actual stress (actual pain).

^d^MDMQ, Multidimensional-Mood-Questionnaire; positive mean change values represent an increase in mood, alertness, and calmness.

**Table 4:** Analyses of medium-term changes in outcome measures between the intervention and the control group.

Variables^a^	Δ values		*P* ^b^	Cohen's *f*
	From baseline (day 1) to follow-up (day 8)			
	Intervention group	Control group	
PSS score^c^	−2.1 (±2.2)	+.9 (±3.2)	0.036^*^	0.57
SF-36 main scales^d^				
(i) Physical component	−1.6 (±6.6)	+.2 (±6.4)	0.500	
(ii) Mental component	+3.3 (±4.0)	+3.1 (±3.6)	0.923	

^a^All continuous data are presented as mean (SD).

^b^ *P* values refer to Mann-Whitney test. ^*^ *P* > 0.05; CI 95%.

^c^PSS, Perceived Stress Scale; positive mean change values represent an increase in perceived stress.

^d^SF-36, Medical Outcomes Study 36-Item Short-Form Health Survey; positive mean change values represent an increase in health related quality of life.

**Table 5:** Analyses of short-term changes in outcome measures between the intervention and the control group.

Variables^a^	Δ values		*P* ^ b^	Cohen's *f*
	Intervention group before and after WATSU	Control group before and after 2 hours waiting period		
VAS stressfulness (mm)^c^				
(i) At day 1	−21 (±22)	−3 (±11)	0.090	
(ii) At day 4	−14 (±14)	+1 (±12)	0.021^*^	0.61
VAS pain (mm)^c^				
(i) At day 1	−8 (±9)	−1 (±4)	0.037^*^	0.51
(ii) At day 4	−11 (±11)	+3 (±10)	0.005^*^	0.72
MDMQ mood scale score^d^				
(i) At day 1	+1.8 (±1.9)	+0.5 (±2.51)	0.175	
(ii) At day 4	+4.7 (±6.2)	−0.1 (±1.13)	0.013^*^	0.56
MDMQ alertness scale score^d^				
(i) At day 1	+2.1 (±3.6)	+0.1 (±1.2)	0.200	
(ii) At day 4	+5.7 (±4.7)	+0.4 (±1.2)	0.001^*^	0.79
MDMQ calmness scale score^d^				
(i) At day 1	4.6 (±3.0)	−0.3 (±1.9)	0.002^*^	0.96
(ii) At day 4	5.6 (±5.8)	−0.5 (±2.2)	0.008^*^	0.71

^a^All continuous data are presented as mean (SD).

^b^ *P* values refer to Mann-Whitney test. ^*^ *P* > 0.05; CI 95%.

^c^VAS, Visual Analog Scale; positive mean change values represent an increase in actual stress (actual pain).

^d^MDMQ, Multidimensional-Mood-Questionnaire; positive mean change values represent an increase in mood, alertness, and calmness.

**Table 6:** Results from analysis of qualitative data reflecting participants' perception of the intervention.

Categories	Mentions (*n* = 60)		Sample phrases
	In absolute values	In % values	
(1) Overall impression	12	21.1	
(1.1) Overall relaxation	8	14.0	*“Total relaxation!” *
(1.2) Overall positive impression	4	7.0	*“Very pleasant!” *

(2) Body related	10	17.5	
(2.1) Physical relaxation	4	7.0	*“I think my belly relaxed too.” *
(2.2) Mobilization	2	3.5	*“My lower back and the right side of the trunk are loosened.” *
(2.3) Transfer to daily life	2	3.5	*“Walking became easier.” *
(2.4) Weightlessness	1	1.8	*“It is great to feel light and weightless once in a while during pregnancy!” *
(2.5) Reduction of pain	1	1.8	*“Less pain.” *

(3) Mind related	15	26.3	
(3.1) Perception	6	10.5	*“No distractions since ears were underneath the surface.” *
(3.2) Peace of mind	4	7.0	*“Free of thoughts and fears.” *
(3.3) Learning	3	5.3	*“Ideal preparation for birth.” *
(3.5) Focus on breech position	2	3.5	*“My child moved a lot but did not turn.” *

(4) Child related	10	17.5	
(4.1) Child relaxed	2	3.5	*“After a while, my child relaxed with me.” *
(4.2) Child active	5	8.8	*“I felt my child moving a lot.” *
(4.3) Interpretation of child's feeling	1	1.8	*“The baby liked it too; she was very quiet.” *
(4.4) Contact to child	2	3.5	*“I think it is a good way to come close to the child—both are in water.” *

(5) Further suggestions	10	17.5	
(5.1) Improvements	4	7.0	*“Start WATSU earlier in pregnancy.” *
(6.1) Emphasizing satisfaction	6	10.5	*“There was nothing I disliked; everything was just great.” *
